# Kinetically Limited Phase Formation of Pt-Ir Based Compositionally Complex Thin Films

**DOI:** 10.3390/ma13102298

**Published:** 2020-05-16

**Authors:** Aparna Saksena, Dimitri Bogdanovski, Hrushikesh Sahasrabuddhe, Denis Music, Jochen M. Schneider

**Affiliations:** 1 Materials Chemistry, RWTH Aachen University, 52056 Aachen, Germany; bogdanovski@mch.rwth-aachen.de (D.B.); music@mch.rwth-aachen.de (D.M.); schneider@mch.rwth-aachen.de (J.M.S.); 2 Department of Metallurgical and Material Science, Indian Institute of Technology, Bombay, Mumbai 400076, India; hrushikesh_sahasrabuddhe@iitb.ac.in

**Keywords:** phase formation, high entropy alloy (HEA), compositionally complex alloys, HEA design criteria, activation energy for surface diffusion

## Abstract

The phase formation of PtIrCuAuX (X = Ag, Pd) compositionally complex thin films is investigated to critically appraise the criteria employed to predict the formation of high entropy alloys. The formation of a single-phase high entropy alloy is predicted if the following requirements are fulfilled: 12 J∙K^−1^ mol^−1^ ≤ configurational entropy ≤ 17.5 J∙K^−1^ mol^−1^, −10 kJ∙mol^−1^ ≤ enthalpy of mixing ≤ 5 kJ∙mol^−1^ and atomic size difference ≤ 5%. Equiatomic PtIrCuAuX (X = Ag, Pd) fulfill all of these requirements. Based on X-ray diffraction and energy-dispersive X-ray spectroscopy data, near-equiatomic Pt_22_Ir_23_Cu_18_Au_18_Pd_19_ thin films form a single-phase solid solution while near-equiatomic Pt_22_Ir_23_Cu_20_Au_17_Ag_18_ thin films exhibit the formation of two phases. The latter observation is clearly in conflict with the design rules for high entropy alloys. However, the observed phase formation can be rationalized by considering bond strengths and differences in activation energy barriers for surface diffusion. Integrated crystal orbital Hamilton population values per bond imply a decrease in bond strength for all the interactions when Pd is substituted by Ag in PtIrCuAuX which lowers the surface diffusion activation energy barrier by 35% on average for each constituent. This enables the surface diffusion-mediated formation of two phases, one rich in Au and Ag and a second phase enriched in Pt and Cu. Hence, phase formation in these systems appears to be governed by the complex interplay between energetics and kinetic limitations rather than by configurational entropy.

## 1. Introduction

Platinum and its alloys are known for their spectacular performance in most corroding environments at elevated temperatures [1,2]. These materials are extensively used in applications demanding corrosion resistance [1,3] as well as high strength such as organic chemical synthesis [4,5], electrode materials in fuel cells [6,7], implantable medical devices [8,9] or precision glass molding [10,11,12,13]. While addition of Au to Pt-based alloys improves the high-temperature corrosion resistance [2], Ir additions enhance the stiffness [1,3]. Elemental Ir is known for its high elastic modulus of 525 GPa which is comparable to some of the existing ceramics in use [3].

As for noble metal senary systems, Sohn et al. [14] investigated the phase formation of two compositions with identical configurational entropies, namely PtPdRhIrCuNi and AuPdAgPtCuNi, by casting. While PtPdRhIrCuNi formed a single-phase, AuPdAgPtCuNi formed a two-phase mixture [14]. The authors attribute the formation of the second phase to energetics, namely the large pairwise enthalpy of mixing [14].

Enhanced properties are one of the key motivations for research on high entropy alloys (HEA) and compositionally complex alloys (CCA) [15]. Miracle and Senkov [16] stated that “HEAs have been tightly associated with finding single-phase solid solutions by controlling configurational entropy”. Furthermore, and in line with a proposal by Miracle and Senkov [16], we refer to multi-principal element alloys forming multiphase microstructures as complex concentrated alloys (CCAs). Contrary to the conventional alloys, HEAs are defined to contain five to 13 major constituting elements where the concentration of each element ranges from 5 to 35 at.% [17]. According to Zhang et al., the formation of HEAs is predicted for materials exhibiting 12 J K^−1^∙mol^−1^ (≈ 1.44∙*R*) ≤ configurational entropies ≤ 17.5 J∙K^−1^ mol^−1^ (≈ 2.10 *R*), −10 kJ∙mol^−1^ ≤ enthalpies of mixing ≤ 5 kJ∙mol^−1^ and atomic size differences ≤ 5% [18], with *R* as the gas constant (≈ 8.3144 J K^−1^∙mol^−1^). However, over time, these guidelines were revealed not to be entirely successful at predicting the formation of single-phase solid solutions [14,19,20,21,22].

While the role of kinetics associated with the synthesis process and its effect on the phase formation has not been investigated for noble metal multi-principal element alloys, a phase formation study on Pt-X (X = Ir, Au) elucidates the role of kinetics during vapor phase condensation [23]. Pt-Ir formed a single metastable fcc phase, while Pt-Au formed two fcc phases. This was attributed to the activation energy for surface diffusion, which is up to six times higher for Ir on a Pt-Ir surface compared to Au on a Pt-Au surface [23].

In the present investigation, the above discussed HEA design criteria [18] are critically appraised for the phase formation of PtIrCuAuX (X = Ag, Pd) thin films. Specifically, the influence of activation energy barriers for surface diffusion on the phase formation is investigated. 

## 2. Experimental and Theoretical Methods

### 2.1. Experimental Methods

A schematic representation of the combinatorial magnetron sputtering setup is presented in previous studies [23,24]. During sputtering the cooling rate was estimated to be > 10^10^ K s^−1^, reaching 10^15^ K∙s^−1^, by Barbee et al. [25]. PtIrCuAuAg thin films were synthesized at substrate temperatures of 520 °C, while PtIrCuAuPd thin films were additionally deposited at 800 °C. All growth experiments were carried out by direct current (DC) magnetron sputtering in an Ar atmosphere at a pressure of 0.4 Pa. The base pressure before deposition was ≤ 7 × 10^−5^ Pa. For the quinary material system, an Au-Cu alloy target (50 at.% each, 99.9% purity) was employed together with the elemental targets of Pt (99.99% purity), Ir (99.9% purity), Ag (99.99% purity) (in the case of PtIrCuAuAg) and Pd (99% purity) (in the case of PtIrCuAuPd). The average applied power densities were 3.6 W∙cm^−2^ for Ir and 2.3 W∙cm^−2^ for Pt, while for Au-Cu alloy target and Ag the power densities were varied between 1.0 and 2.5 W∙cm^−2^ to enable the synthesis of quinary PtIrCuAuAg thin films. For the PtIrCuAuPd thin film depositions, the average power densities were 3.6 W∙cm^−2^ for Ir and 2.3 W∙cm^−2^ for Pt, while the Au-Cu alloy target and Pd were subjected to 3.6 and 1.0 W∙cm^−2^, respectively. The substrate-to-target distance was 10 cm for all growth experiments, whereby Al_2_O_3_ (0001) single crystal substrates were used without substrate rotation.

The composition gradients were analyzed by energy-dispersive X-ray spectroscopy (EDS) using a JEOL JSM-6480 scanning electron microscope (JEOL Ltd., Tokyo, Japan), equipped with an EDAX Genesis 2000 detector (EDAX Inc./ Ametek MAD, Mahwah, NJ, USA). The elemental compositions were determined by ZAF corrections [26] utilizing the measured characteristic X-ray intensities of the L lines for Pt, Ir, Au Ag and Pd, and the K line for Cu. The acceleration voltage was 15 keV.

Atom probe tomography (APT) was used to elucidate the spatially resolved composition via local electrode atom probe (LEAP 4000X HR^TM^ Cameca Instrument, Ametek MAD, Mahwah, NJ, USA). The laser-assisted field evaporation measurements were performed at −213 °C with a laser energy of 80 pJ and a frequency of 125 kHz. The IVAS 3.8.0 software package was used for data reconstruction and analysis.

Structure analysis of the as-deposited thin films was performed using X-ray diffraction (XRD). A Bruker D8 general area detection diffraction system (GADDS, Bruker, Billerica, MA., USA) in grazing incidence geometry was employed to perform 2*θ* scans at a grazing angle of 10˚, with Cu Kα (λ = 1.5406 Å) radiation. The generator voltage and current were set to 40 kV and 40 mA, respectively.

### 2.2. Theoretical Methods: Ab Initio Calculations

For PtIrCuAuX (X = Ag, Pd), first-principles calculations based on density-functional theory (DFT) were performed employing the Vienna Ab Initio Simulation Package (VASP, version 5.4.4, Universität Wien, Vienna, Austria) [27,28]. The projector-augmented wave (PAW) method [29] was used for basis set representation within the framework of the generalized gradient approximation (GGA) with the established functional by Perdew, Burke and Ernzerhof (PBE) to account for exchange and correlation [30,31]. The plane-wave cut off energy and the energetic convergence criterion were set to be 500 and 10^−6^ eV, respectively. The Brillouin zone integration was performed via Blöchl’s tetrahedron method for plane-wave basis sets [32], utilizing an appropriate *k*-points grid of 3 × 3 × 1, chosen to ensure energetic convergence and generated via the Monkhorst–Pack approach [33]. As the system in question is not magnetically active, spin polarization was not considered in the calculations. The structures were fully relaxed in terms of cell volume and atomic positions. The total ground state energy of the equilibrium structure was obtained from an additional static calculation.

The energy of formation of the quinary systems was calculated with the 108-atom special quasi-random structures (SQS) [34]. The Warren–Cowley short-range order parameter [35] was within 0.1 for 8 coordination shells. The average volume of the relaxed SQS cell was used to obtain the lattice constants of different configurations of PtIrCuAuX (X = Ag, Pd) by fitting the total energy as a function of cell volume using the Birch–Murnaghan equation of state [36].

The activation energy barrier for surface diffusion was estimated according to Chang et al. [34], by moving an atom stepwise from its equilibrium lattice site to the nearest neighboring site in the close-packed plane (111) along the <110> direction. The movement of the atom occurs within the surface layer (no adatom motion) to a neighboring vacancy to mimic the sputtering process. This method has been used to investigate the influence of surface diffusion on the phase formation of Cu-W [34,37], Cu-V [37], Pt-X (X = Ir, Au) [23] and TiAlN [38] thin films. The activation energy barrier was determined by the difference of the maximum (saddle point) and minimum (equilibrium) energies along this path. These calculations were carried out for a 180-atom supercell whose energy per atom is closest to that of an SQS cell with identical composition. The thickness of the vacuum layer was 10 Å. It should be stressed that, firstly, all these calculations were performed for the movement of an atom to a neighboring site which may or may not be the respective global minimum for a given system, and secondly, that the activation energy barrier of surface diffusion can also depend on the environment of the moving atom.

Bonding interaction analyses were performed utilizing the crystal orbital Hamilton population (COHP) method, with local orbitals projected from delocalized plane-wave basis sets [39,40] as implemented in the LOBSTER package (version 3.2.0, Chair of Solid State Chemistry, Institute of Inorganic Chemistry, RWTH Aachen University, Aachen, Germany) [41,42]. Two structural models of a slightly Pt/Ir-enriched 108-atom configuration, Pt_23_Ir_23_Cu_21_Au_21_X_20_ (X = Ag, Pd), as generated via the SQS approach described above, with identical atomic arrangement and solely differing in the atomic species of X, were used in order to isolate the sole effect of atomic substitution on bonding. After full relaxation of these two systems, the generated plane-wave wave functions were projected onto local atomic orbitals to enable the quantification of the integrated COHP (ICOHP) values at the Fermi level for all possible atom pair interactions within the first three coordination shells (CS) of a given atom. These ICOHP values serve as an indirect but strongly correlated, and thus reliable, descriptor of the respective bond strength; however, by nature of the method, covalent bonding is assumed. Thus, in metallic systems with a high degree of electron delocalization, bonding characteristics are only partially described by the COHP approach. Nevertheless, trends are regularly reproduced well, and COHP analyses are routinely used for the qualitative description of bonding in metallic systems in literature [43,44,45].

## 3. Results and Discussion

**Figure 1:**
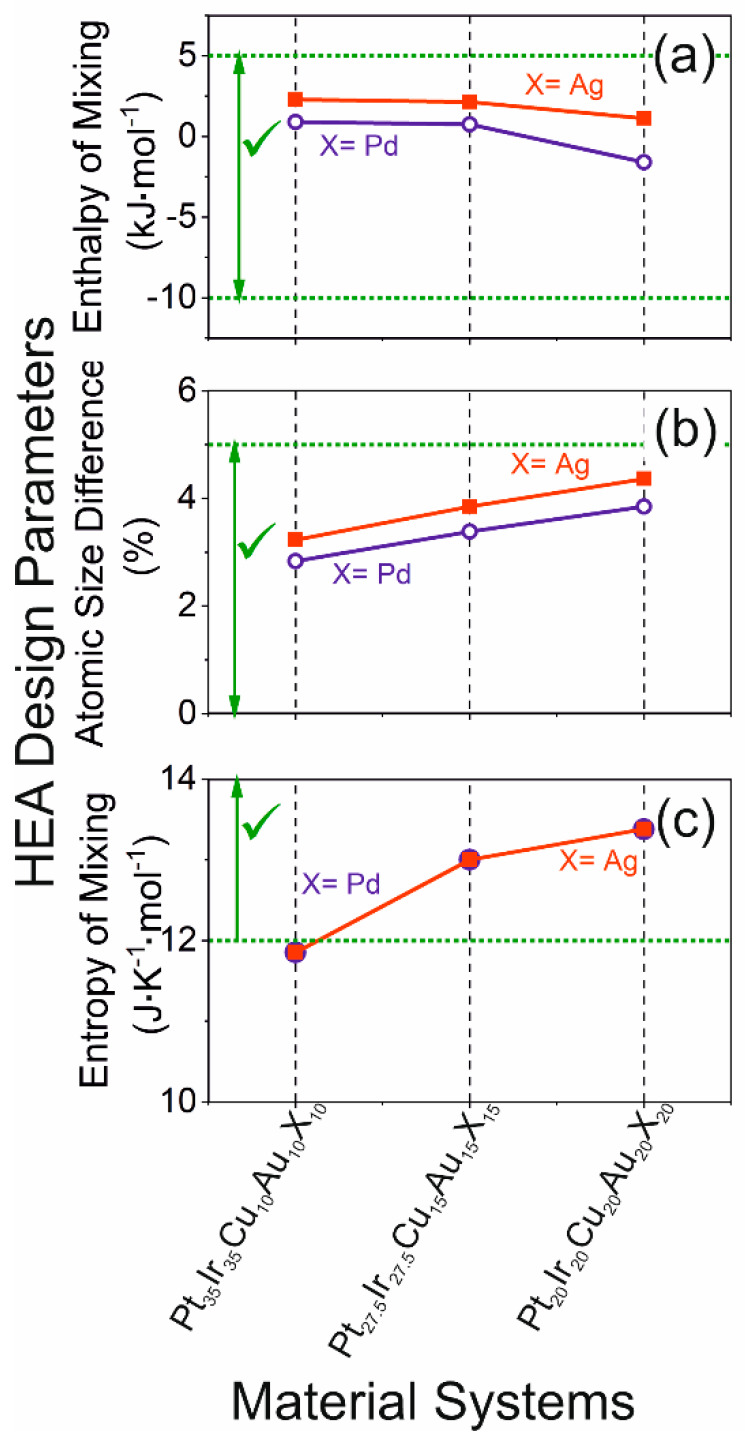
The enthalpy of mixing (**a**), the entropy of mixing (**c**), atomic size (**b**) difference for the different configurations of PtIrCuAuX (X = Ag, Pd). The regions marked with “**✓**” fulfill the high entropy alloy (HEA) design criteria. The data points are connected to guide the eye.

According to Zhang et al. [18], the formation of a single-phase solid solution is predicted based on the following criteria: atomic size difference (∂ (%)), enthalpy of mixing (∆*H*_mix_) and the configurational entropy (∆*S*_mix_) of the HEA. Specifically, for the formation of a single-phase HEA, the following criteria must be fulfilled: −10 kJ∙mol^−1^ ≤ ∆*H*_mix_ ≤ 5 kJ∙mol^−1^ [18], ∂ ≤ 5% [18] and 12 J K^−1^ mol^−1^ (≈ 1.44∙*R*) ≤ ∆*S*_mix_ ≤ 17.5 J∙K^−1^ mol^−1^ (≈ 2.10∙*R*) [18]. These criteria are fulfilled for the Pt_20_Ir_20_Cu_20_Au_20_X_20_ and Pt_27.5_Ir_27.5_Cu_15_Au_15_X_15_ (X = Ag, Pd) compositions, whereas Pt_35_Ir_35_Cu_10_Au_10_X_10_ does not fulfill 12 J∙K^−1^∙mol^−1^ (≈ 1.44 *R*) ≤ ∆*S*_mix_, as shown in Figure 1. Hence, the formation of a single solid solution phase is predicted for Pt_20_Ir_20_Cu_20_Au_20_X_20_ and Pt_27.5_Ir_27.5_Cu_15_Au_15_X_15_ (X = Ag, Pd).

**Figure 2:**
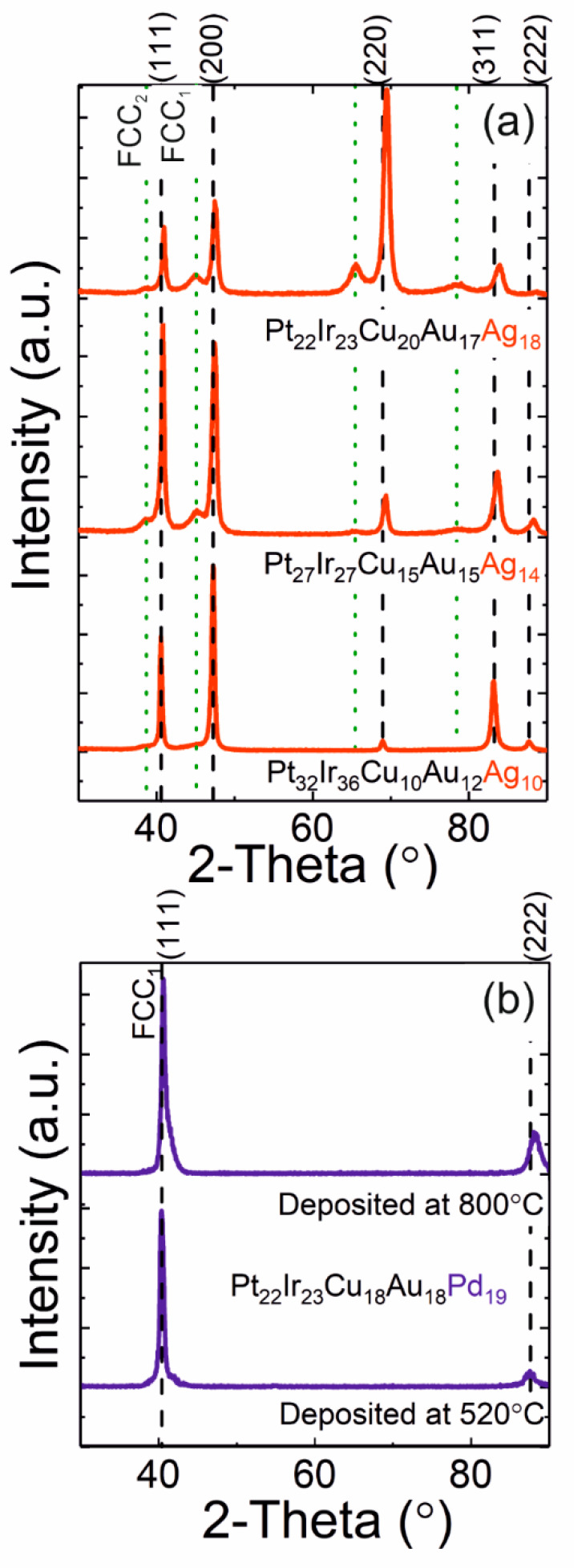
X-ray diffraction patterns depicting phase formation behavior of the PtIrCuAuAg (**a**) and PtIrCuAuPd (**b**) (X = Ag, Pd) material systems. FCC_2_ designates a secondary fcc phase formation.

Thin films with compositions similar to the above-discussed reference compositions were deposited. Figure 2a shows the XRD pattern of the near-equiatomic Pt_22_Ir_23_Cu_20_Au_17_Ag_18_ thin film, which, despite possessing a configurational entropy of 13.30 J∙K^−1^∙mol^−1^ (≈ 1.60∙*R*), clearly shows the formation of two distinct fcc phases where the lattice parameters of FCC_1_ and FCC_2_ are 3.83 and 4.03 Å, respectively. Hence, the observed phase formation is in conflict with the prediction discussed in the context of Figure 1. Pt_27_Ir_29_Cu_15_Au_15_Ag_14,_ exhibiting a configurational entropy of 12.94 J K^−1^ mol^−1^ (≈ 1.55∙*R*), also formed two fcc phases where, correspondingly, the lattice parameters of FCC_1_ and FCC_2_ are 3.83 Å and 4.02 Å, respectively. However, as the Ag, Au and Cu concentrations were reduced, the intensity of FCC_2_ weakens and the phase formation in Pt_32_Ir_36_Cu_10_Au_12_Ag_10,_ exhibiting a configurational entropy of 12.03 J K^−1^ mol^−1^ (≈ 1.45∙*R*), is dominated by FCC_1_ with a lattice parameter of 3.83 Å, as observed by XRD, see Figure 2a. In Figure 2b, the diffractogram of a Pt_22_Ir_23_Cu_18_Au_18_Pd_19_ thin film deposited at 520 °C substrate temperature with a configuration entropy of 13.34 J∙K^−1^∙mol^−1^ (≈ 1.60 *R*) clearly suggests the formation of a single fcc phase with a lattice parameter of 3.87 Å, in line with the above-discussed phase prediction guideline. However, the asymmetric (111) peak in the diffractogram of a film deposited at 800 °C substrate temperature, with an identical composition, clearly indicates the formation of a second phase absent at lower temperatures. If the phase formation were governed by configurational entropy, the more negative *T*d*S* term should favor the formation of a single-phase. Hence, the phase formation observed herein is inconsistent with the predictions based on the aforementioned HEA design criteria.

**Figure 3:**
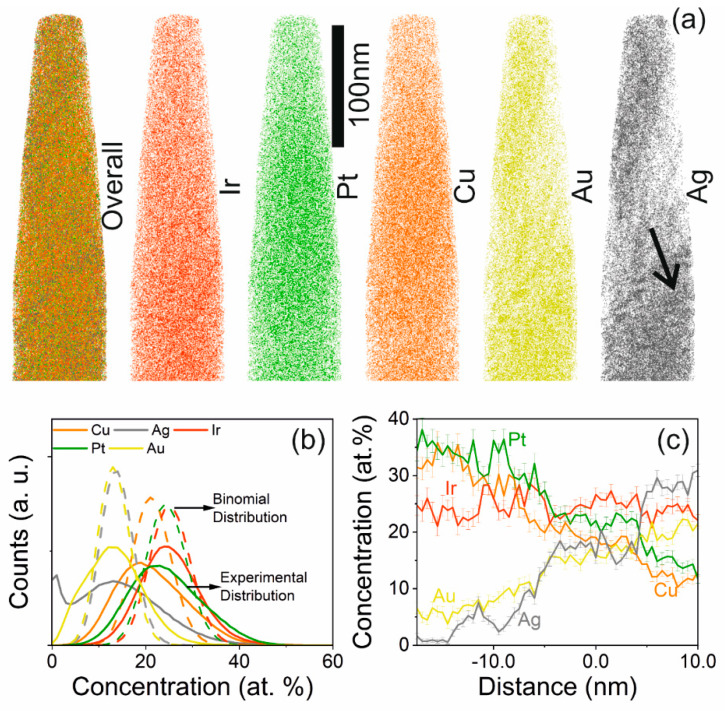
(**a**) Depiction of the overall as well as individual elemental distribution of Pt_22_Ir_23_Cu_20_Au_17_Ag_18_, via atom probe tomography. (**b**) Frequency distribution analysis of the constituting elements. (**c**) The one-dimensional concentration profile along the direction highlighted in (**a**), depicting the 2 phases: Au-Ag-depleted and -rich regions.

To analyze the local chemical composition of the two phases, identified by XRD in the Pt_22_Ir_23_Cu_20_Au_17_Ag_18_ thin film deposited at 520 °C, APT was performed. Figure 3a shows the overall as well as the individual elemental distribution of the constituting species. It is evident that Ag and Au are not randomly distributed. In a frequency distribution analysis, a binomial or random distribution of the elements would result in a Pearson’s coefficient (*μ*) equal to zero [46]. As shown in Figure 3b, all of the elements exhibit a non-random distribution. The calculated Pearson’s coefficients for Pt (*μ* = 0.96), Cu (*μ* = 0.96), Au (*μ* = 0.96), and Ag (*μ* = 0.99) are consistent with clustering. The Pearson’s coefficient of Ir (*µ* = 0.86) suggests less clustering as compared to Pt, Cu, Au and Ag. A one-dimensional concentration profile for a cylindrical region of interest of 10 × 10 × 40 nm^3^ is compiled along the direction marked by the black arrow in Figure 3a and is shown in Figure 3c. From left to right, the analyzed region transitions from a phase, depleted in Ag and Au, to the second phase, depleted in Pt and Cu. The Ir concentration is similar in both phases. In the Pt-Cu rich phase, Au and Ag content are depleted by 12 and 15 at.%, respectively, compared to the integral composition where Pt and Cu are both enriched by 15 at.%. On the other hand, the Au-Ag rich region exhibits a depletion of both Pt and Cu by 8 at.% each from the integral composition, as determined by APT.

**Figure 4:**
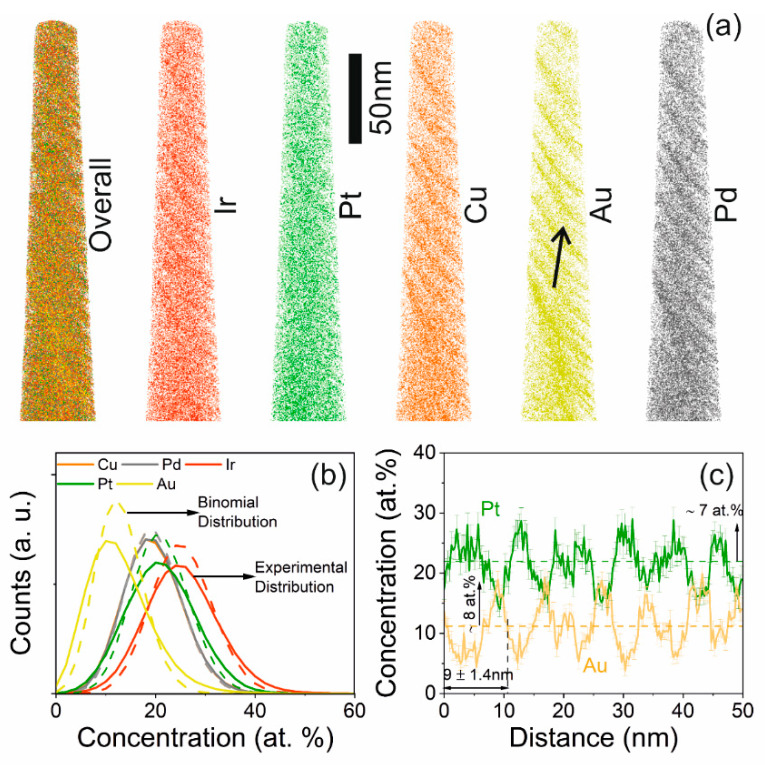
(**a**) A depiction of the overall elemental distribution as well as individual elemental distribution of Pt_22_Ir_23_Cu_18_Au_18_Pd_19_ deposited at 520 °C substrate temperature, via atom probe tomography. (**b**) Frequency distribution analysis of the respective constituting elements. (**c**) One-dimensional concentration profile along the direction highlighted in (**a**), depicting the chemical modulation of Au and Pt.

Figure 4a shows the overall as well as the individual elemental distribution of a Pt_22_Ir_23_Cu_18_Au_18_Pd_19_ thin film deposited at 520 °C substrate temperature, which according to XRD contains a single fcc phase. Although the overall elemental distribution appears quite homogeneous, chemical fluctuations are evident from the individual elemental distribution. The frequency distribution analysis is shown in Figure 4b. The Pearson coefficient of Au (*μ* = 0.71) indicates a non-random distribution. Even though Pt and Ir may appear to be more randomly distributed than Cu and Pd based on the images presented in Figure 4a, a frequency distribution analysis reveals the Pearson’s coefficients of Cu (*μ* = 0.10) and Pd (*μ* = 0.08), clearly indicating a more random distribution than for Ir (*μ* = 0.41) and Pt (*μ* = 0.35). This is consistent with the one-dimensional concentration profile along the direction marked by the black arrow in Figure 4a, for a cylindrical region of interest of 10 × 10 × 50 nm^3^ as shown in Figure 4c, clearly indicating anti-correlated fluctuation between Pt and Au with a wavelength of 9 ± 1 nm. Au content varies from 12 ± 7 at.%, while Pt varies between 22 ± 7 at.%.

The phase formation observed for both Pt_22_Ir_23_Cu_20_Au_17_Ag_18_ and Pt_22_Ir_23_Cu_18_Au_18_Pd_19_ thin films deposited at 520 °C clearly contradicts the predictions based on above-discussed requirements for the formation of single-phase HEA: Pt_22_Ir_23_Cu_20_Au_17_Ag_18_ forms two fcc phases, while Pt_22_Ir_23_Cu_18_Au_18_Pd_19_ forms a metastable fcc phase with chemical modulation at the nm scale. At a growth temperature of 800 °C, the onset of the formation of a second phase was observed by XRD for Pt_22_Ir_23_Cu_18_Au_18_Pd_19_ thin films. In an effort to rationalize it, the disparity between the measured and predicted phase formation serves as the motivation to investigate the effect of kinetics on the phase formation during sputtering of PtIrCuAuX (X = Ag, Pd) thin films. Specifically, the magnitude of the activation energy barrier for surface diffusion of the individual species and the associated bond strength trends are investigated.

**Figure 5:**
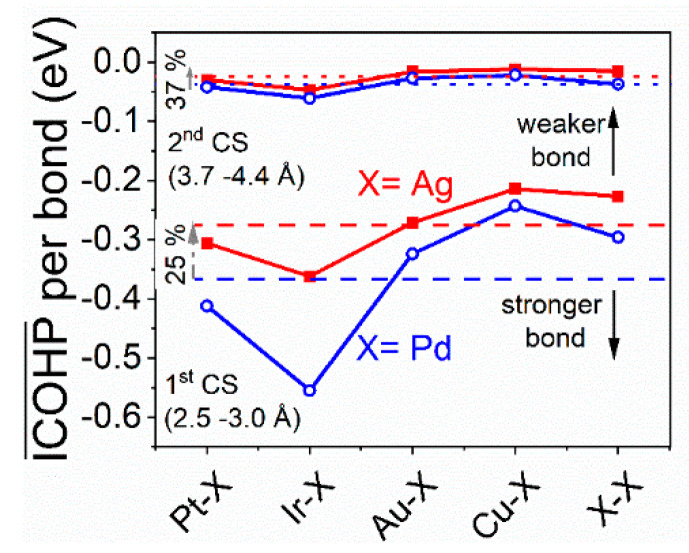
Integrated crystal orbital Hamilton population (ICOHP) per bond for binary combinations of X (X = Ag, Pd) with Au, Cu, Ir, Pt and X itself in a near-equiatomic PtIrCuAuX (X = Ag, Pd) system. CS signifies the coordination shell and the dashed lines depict the average ICOHP per bond across all interactions for the respective systems.

Average ICOHP values per bond, directly correlated with the (not inherently quantifiable) bond strength, are calculated for the heteroatomic interactions of X (X = Ag, Pd) with Pt, Ir, Cu, Au and the homoatomic X–X interaction, as shown in Figure 5. In general, interactions of a given atom with atoms in its first coordination shell (CS) and its second CS experience an average shift of 25% and 37%, respectively, of the average ICOHP per bond towards more positive values, and thus weaker bonds, upon substitution of Pd with Ag. The average ICOHP per bond for interactions with atoms in the first CS is thus strongly influenced by the substitution of Pd with Ag, while being much less numerically affected by the substitution for interactions with atoms in the second CS; while the relative shift (37%) is higher, the absolute values of the average ICOHP in the second CS are lower by an order of magnitude, leading to only marginal absolute differences. With an average relative shift of <20% and comparable absolute values to those in the second CS, there is no quantifiable impact on the ICOHP values for interactions with atoms in the third CS as a result of the substitution. Thus, this type of interaction is not shown. Recalling that the ICOHP value is an indirect but qualitatively accurate descriptor for the bond strength, we summarize that the average strength of bonds formed between Ag and all other constituting elements is significantly weaker than that of bonds formed between Pd and other elements. The implications thereof for surface diffusion are discussed next.

**Figure 6:**
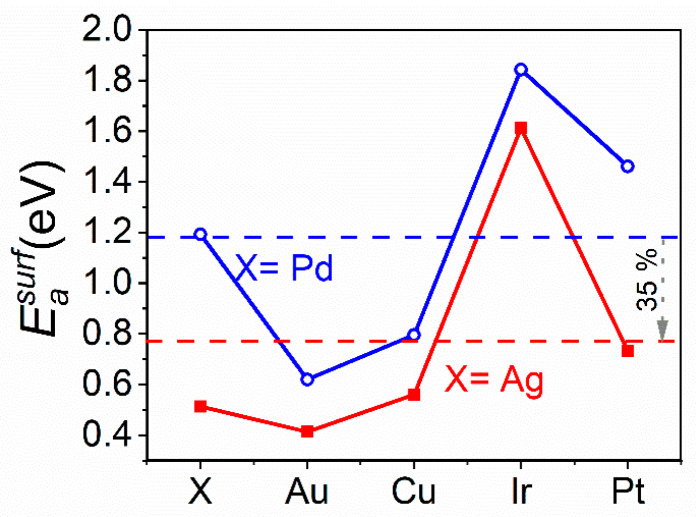
Activation energy barrier for surface diffusion ($E_{a}^{surf}$), obtained via ab initio calculations for Ag, Au, Cu, Ir, Pd and Pt on the (111) surface in <110> direction of the equiatomic PtIrCuAuX (X = Ag, Pd) material system. The dashed line depicts the average activation energy barrier for the respective material systems.

The activation energy barrier for surface diffusion of all the five constituting elements was calculated for both equiatomic PtIrCuAuAg and PtIrCuAuPd. These configurations closely mimic the experimental compositions. Each element is moved to its neighboring vacancy site in the (111) surface plane in <110> direction. Figure 6 shows the difference between the lowest and highest energies during this process for each element in the discussed equiatomic composition, a comparison of the activation energy barriers for surface diffusion of equiatomic PtIrCuAuAg and PtIrCuAuPd is hence, illustrated. It is evident that substituting Pd with Ag causes a 35% decrease in the average activation energy barrier for surface diffusion, corresponding to a higher mobility of elements in PtIrCuAuAg and in line with the decreased bond strengths. Although these material systems possess identical configurational entropies, the higher activation energy barriers lead to the formation of a kinetically limited single metastable phase in Pt_22_Ir_23_Cu_18_Au_18_Pd_19_ thin films, compared to the 35% lower average activation energy barriers enabling the formation of two fcc phases in Pt_22_Ir_23_Cu_20_Au_17_Ag_18_ thin films. Hence, it is now evident that the phase formation in PtIrCuAuX (X = Ag, Pd) appears to be governed by a complex interplay between energetics and kinetic considerations and cannot be predicted based on the above-discussed criteria for the formation of single-phase HEAs.

## 4. Conclusions

The phase formation of PtIrCuAuX (X = Ag, Pd) thin films was studied on various length scales. Both compositions fulfill the criteria for the formation for single-phase high entropy alloys, namely 12 J∙K^−1^∙mol^−1^ ≤ configurational entropy ≤ 17.5 J∙K^−1^ mol^−1^, −10 kJ∙mol^−1^ ≤ enthalpy of mixing ≤ 5 kJ∙mol^−1^ and atomic size difference ≤ 5%. While near-equiatomic Pt_22_Ir_23_Cu_18_Au_18_Pd_19_ thin films, based on XRD, crystallize in a single fcc phase, APT studies revealed anti-correlated, local chemical modulations of Au and Pt with 12 ± 7 and 22 ± 7 at.%, respectively, and a wavelength of 9 ± 1 nm.

For near-equiatomic Pt_22_Ir_23_Cu_20_Au_17_Ag_18_ thin films, the formation of two fcc phases was observed by XRD. APT revealed that one phase was rich in Au and Ag, while the other one was rich in Pt and Cu. Furthermore, the solubility of both Au and Ag in the Pt-Cu- rich region was observed to be ≥ 5 and ≥ 3 at.%, respectively, while the solubility of Pt and Cu in the Au-Ag rich region is ≥ 10 at.%. These observations are clearly inconsistent with the HEA design criteria discussed above.

In an attempt to rationalize the disparity between experimentally obtained data and the phase formation predictions, the bond strengths and the activation energy barriers for surface diffusion of each constituting element were calculated. The effect of substitution of Pd by Ag on the bond strength was probed via the average integrated crystal orbital Hamiltonian population per bond. Bond strength for heteroatomic interactions of X-Y (X = Ag, Pd, Y = Pt, Ir, Cu, Au) decreases on average by 25% in the first coordination shell. This decrease in bond strength causes a concomitant decrease in the average surface diffusion activation energy barrier by 35%. These observations can aid in rationalizing the phase formation in these material systems, as the larger energetic barriers for Pt_22_Ir_23_Cu_18_Au_18_Pd_19_ enable the formation of a single, metastable phase at a substrate temperature of 520 °C due to kinetically limited growth. In contrast, the barriers for Pt_22_Ir_23_Cu_20_Au_17_Ag_18_, lower by 35% on average, result in sufficient mobility to initiate phase separation at the same substrate temperature. It is hence evident that the phase formation of physical vapor deposited PtIrCuAuX (X = Ag, Pd) thin films is defined by a complex interplay of energetics and kinetic limitations and not by the configurational entropy.
